# A real-world framework for automated product recognition and catalog generation: dataset, model, and analysis

**DOI:** 10.1038/s41598-026-42266-9

**Published:** 2026-05-12

**Authors:** Mayank Sah, Jimson Mathew, P. Dayananda

**Affiliations:** 1 Department of Computer Science and Engineering, Indian Institute of Technology, Patna, India; 2 Manipal Institute of Technology Bengaluru, Manipal Academy of Higher Education, Manipal, Karnataka, India

**Keywords:** Grocery Identification, Deep Neural Network, Object Recognition, Dataset, Omni-Scale features, Lightweight model, Engineering, Mathematics and computing

## Abstract

The identification and management of grocery items in retail environments have traditionally relied on barcode-based systems, which require significant human intervention and underutilize existing surveillance infrastructure. Computer vision–based approaches offer a promising alternative for automated product recognition. However, many existing grocery datasets remain relatively homogeneous or limited in scale, geographic diversity, or real-world variability. To support more realistic evaluation settings, we present a large-scale grocery dataset collected from eight stores across multiple states in India. The dataset comprises over 13,000 images spanning 349 product categories and captures practical retail challenges such as dense shelf arrangements, occlusions, viewpoint variations, and visual ambiguity. Rather than claiming novelty in addressing these challenges individually, our contribution lies in systematically integrating them within a unified and diverse dataset framework. We also introduce a lightweight product identification pipeline based on omni-scale feature learning, designed to balance representational capacity and computational efficiency. The proposed model achieves a mAP@0.50 of 58.3, a precision of 72.9%, and a recall of 77.9% on the proposed dataset, demonstrating competitive performance while maintaining a compact architecture. Comprehensive comparisons with established benchmark models further contextualize our contributions within the broader literature. Overall, this work provides a diverse evaluation benchmark and an efficient detection framework for practical retail deployment.

## Introduction

Object detection has remained a core research area within computer vision, with its progress significantly accelerated by advances in deep learning. Over the past decade, the field has transitioned from traditional handcrafted feature-based techniques, such as SIFT^1^ and HOG^2^-to highly optimized deep learning architectures. This shift began with the introduction of two-stage detectors, most notably R-CNN^3^, and later evolved toward faster and more efficient single-stage detection frameworks including YOLO^4^, YOLOv3^5^, YOLOv5^6^, YOLOv8^7^, YOLOv9^8^, YOLOv10^9^, YOLOv11^10^, YOLO26^11^ and RetinaNet^12^. More recent developments have introduced transformer-based architectures such as DETR^13^ and RT-DETR^14^, which leverage attention mechanisms to enable end-to-end detection pipelines with improved scalability and real-time performance. A common requirement across these state-of-the-art detection models is access to large-scale, diverse, and well-annotated training datasets. Benchmark datasets such as ImageNet^15^, CIFAR^16^, COCO^17^, and PASCAL-VOC^18^ have played a pivotal role in shaping detection research by providing the necessary data foundation for learning robust and generalizable visual representations. However, while these datasets have been essential for algorithmic development, their general-purpose nature limits direct applicability to highly specialized real-world domains.

**Fig. 1:**
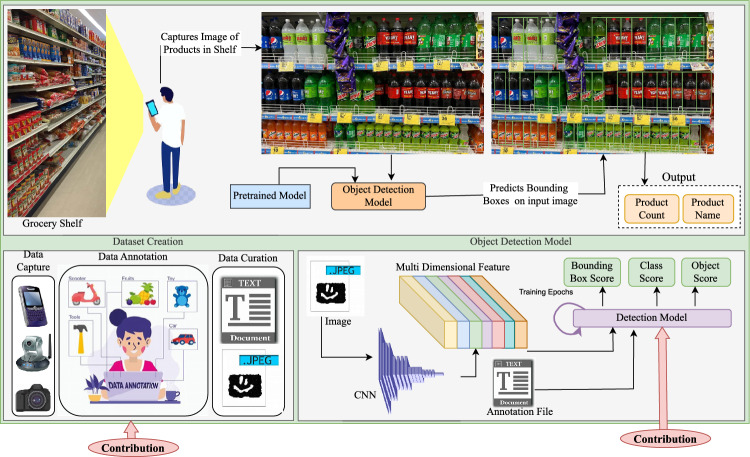
Overview of the real-world application pipeline for grocery product identification using the Grocer-Help dataset. The diagram illustrates a practical scenario where a user captures an image of grocery items on a store rack using a mobile device. This image is processed by the object detection model, which identifies and classifies the products, and outputs both the detected product categories and their respective counts. The figure also highlights the key contributions of this work–the introduction of a new dataset and the development of a novel backbone optimized for accurate and efficient grocery item detection.

Recent research has also explored the integration of Retrieval-Augmented Generation (RAG) frameworks to support object detection tasks^19^. Such approaches leverage external knowledge sources to enrich visual predictions with contextual information. In a related direction, the work in^20^ demonstrates how incorporating contextual cues can help address inherent inconsistencies and uncertainties present in real-world environments. These studies highlight the growing importance of combining visual features with contextual reasoning to improve robustness and reliability in practical deployment scenarios. The deployment of state-of-the-art object detection models across domain-specific applications, such as traffic monitoring^21^, gesture recognition^22^, and unmanned aerial vehicles^23^-has accelerated significantly. With this progress, dense object detection has emerged as a key research focus, particularly in scenarios involving numerous visually similar objects arranged in close proximity^24,25^. Within this context, grocery product identification has evolved into a challenging and specialized application domain. Existing approaches differ in how they conceptualize the task: some frame it as a dense object detection problem^26^, whereas others approach it as a fine-grained image classification task^27^, reflecting the ambiguity and complexity inherent to retail product recognition. Grocery environments present several unique visual challenges, including variations in object scale, packaging design, viewpoint, clutter, occlusion, and illumination. While modern detection architectures achieve strong performance on widely used general-purpose benchmarks, they frequently struggle in such settings, particularly when distinguishing among highly similar product variants or operating in densely packed shelf scenes. Notably, product identification cannot be exclusively treated as either a dense object detection task or a classification task. The distinction depends largely on the image acquisition scenario. When a product image is captured at close range, the task aligns more closely with fine-grained classification; however, when the same product appears at a distance, such as on a supermarket shelf-the task becomes one of dense object detection. This duality introduces additional complexity and highlights the need for models capable of adapting across varying capture conditions. Figure 1 illustrates this continuum, highlighting the full workflow, from mobile-based image capture and dataset construction to model training and deployment. Such systems support a broad range of applications, including automated catalog generation, product retrieval, inventory monitoring, checkout automation, and even product summarization or description generation.

Several domain-focused datasets, such as the Freiburg Groceries dataset^28^ and SKU110K^26^, have contributed valuable resources toward advancing research in retail-focused perception. However, these datasets exhibit notable limitations. Some contain only a narrow set of product categories, while others rely on controlled or homogeneous acquisition setups. Such characteristics limit their ability to capture the complexity and variability of real-world retail scenes, where factors such as shelf distance, viewing angle, lighting conditions, reflections, wear on packaging, and partial visibility strongly influence detection performance. Moreover, the grocery domain is inherently shaped by geographic and cultural context. A dataset built around products prevalent in one region may generalize poorly elsewhere due to differences in brand ecosystems, packaging conventions, and consumer preferences. This challenge is particularly pronounced in countries like India, where regional manufacturers and product assortments vary widely. In response to these needs and to advance research in this direction, we define following research objectives:

- **RO1:** To design and evaluate a novel detection backbone capable of effectively representing and integrating multi-scale object information, enabling robust detection performance across varying object sizes within complex visual scenes.
- **RO2:** To analyze the performance of existing benchmark datasets on state-of-the-art single-stage object detection models, and to evaluate the proposed detection model on these same benchmark datasets for comparative assessment.
- **RO3:** To develop a comprehensive grocery dataset that captures real-world retail variability, including diverse product categories, imaging conditions, and shelf arrangements, and to evaluate its effectiveness by benchmarking state-of-the-art object detection models alongside the proposed approach.
- **RO4:** To assess the robustness of the proposed model across varied image capture scenarios, namely close-shot, long-shot, and online images, provided by the dataset, and to analyze how these variations impact detection performance.
- **RO5:** To investigate the model’s consistency across different store environments, given that the dataset includes images captured from geographically diverse retail stores.
- **RO6:** To study the impact of class instance frequency on detection performance, particularly considering the imbalance in the number of occurrences of product classes due to varied store layouts and locations.

To address the challenges of grocery product identification in real-world retail environments, we introduce a new dataset named Grocer-Help along with a lightweight object detection architecture based on omni-scale feature aggregation. The Grocer-Help dataset is designed to reflect realistic in-store variability by incorporating diverse capture conditions, including varying camera viewpoints, distances (close-range, shelf-level, and long-range), lighting conditions, and retail layouts. A detailed description of the dataset construction and characteristics is provided in Section Proposed dataset. Existing deep learning models often experience degraded performance under such domain variations due to limited scale-awareness and insufficient robustness to visual diversity. To address these limitations, we propose an omni-scale feature aggregation–based detection architecture that enhances multi-scale object representation and improves detection reliability across heterogeneous imaging conditions. The methodology and architectural details of the proposed model are presented in Section Methodology. Experimental evaluation, discussed in Section Experimental details, demonstrates that the proposed model generates more perspective-aware detections and achieves improved performance in terms of precision and recall compared with state-of-the-art baseline models. Furthermore, the diversity of the proposed dataset enables systematic evaluation under challenging scenarios such as domain shifts, distance variation, and class imbalance. The primary research contributions (RC) of this work are summarized as follows:

- **RC1:** We present a novel dataset comprising over 13,000 images of 349 grocery product classes, collected from eight different stores located across five states in India.
- **RC2:** The dataset is designed to capture geographical and environmental diversity, reflecting variations in product availability, brand presence, shelving patterns, camera settings, lighting conditions, and viewing angles, including both close and long-range captures.
- **RC3:** We propose a new omni-scale feature-based object detection model tailored to handle the wide range of feature scales and object densities found in grocery retail imagery.
- **RC4:** We perform a comprehensive baseline evaluation, comparing our proposed model against state-of-the-art object detection algorithms on both existing benchmark datasets and our proposed Grocer-Help dataset, thereby validating the effectiveness and generalizability of both.
- **RC5:** Leveraging the dataset’s diversity, we conduct in-depth scenario-based analyses, including domain shift evaluation (e.g., store-wise and geographic impact), image distance analysis, and training instance distribution, to better understand real-world detection performance under varying conditions.

To the best of our knowledge, no existing dataset comprehensively addresses the combined effects of camera angle variation, shot distance (e.g., close and long-range captures), and geographic diversity in the context of grocery product identification. While prior studies have explored these factors in isolation or as secondary considerations, a holistic analysis integrating all these aspects remains absent from the literature.

The remainder of this paper is organized as follows. Section Literature review presents a detailed review of related work, including existing grocery datasets and object detection models. Section Proposed dataset introduces the proposed Grocer-Help dataset, highlighting its structure and key features. Section Methodology describes the architecture of the proposed omni-scale feature-based object detection model. Section Experimental details provides an overview of the experimental setup and implementation details. Section Results & analysis presents the results and analyses, covering baseline comparisons, dataset generalizability, and diversity assessments. Section Limitations and future work discusses the limitations of this work and outlines potential directions for future research. Finally, Section Conclusion concludes the paper with a summary of the key findings and contributions.

## Literature Review

In this section, we provide a brief overview of existing grocery product datasets. Table 1 summarizes key details of widely used datasets, including the number of images, classes, image types, and resolutions.

**Table 1:** Summary of existing grocery product datasets, highlighting key characteristics including the number of images, number of object classes, image resolution, and notable features. The comparison provides insight into the scope and limitations of each dataset with respect to product variety, visual quality, and applicability to real-world grocery identification tasks.

Name	Classes	Images	Resolution	Description	Comments
GroZi-120^29^	120	5793	100$\times$100	Close shot, Online	Uses online images for training and real-life images for testing. Very low accuracy. No distance/angle variation.
GroZi-3.2K^30^	80	8350	640$\times$640	Close shot, Online	Better accuracy than GroZi-120, but still lacks distance and angle variation.
Freiburg^28^	25	4947	256$\times$256	Close shot	Ideal conditions only. No distance or angle variation. Low intra-class diversity.
SKU110K^26^	1	11762	Varied	Long shot	Focuses only on long-shot images. Used for object detection, not classification.
Webmarket^27^	200	3153	2272$\times$1704 or 2592$\times$1944	Long shot	Designed for object retrieval. Matches product queries to rack images.
Grocery-Store^31^	81	5125	348$\times$348	Close shot, Online	One-to-one product image matching. No support for multi-product detection.
Holoselecta^32^	109	295	3480$\times$4640	Close shot	Focused on vending machine items. No distance or angle variation.
Locount^33^	140	50394	1920$\times$1080	Close shot	Frontal view images only. Focus on object counting. No real-world variation.
**Grocer-Help (Proposed Dataset)**	**349**	**13771**	**Varied**	**Close shot, Online, Long shot**	**Captures real-world distance and angle variations. Enables multi-product identification and robust recognition.**

**Fig. 2:**
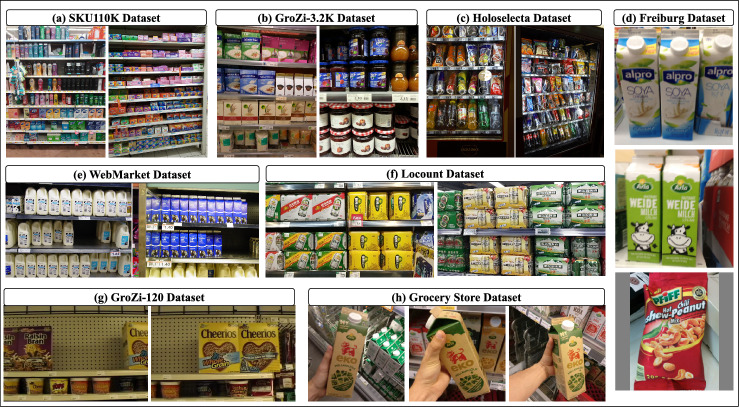
Sample image instances from existing grocery datasets. The figure showcases the diversity in visual characteristics such as product arrangement, image resolution and background clutter found in publicly available datasets used for grocery product identification tasks.

Merler et al.^29^ introduced GroZi-120, a pioneering dataset comprising 120 grocery item categories. The training set consists of 676 in-vitro (web-sourced) images, while the test set includes 11,194 in-situ (real-world store) images, often extracted from video frames. Each training image contains a single item, while test images may contain multiple objects. Despite its innovation, GroZi-120 presents a significant domain gap between the training and testing data, resulting in poor performance when evaluated using modern object detection models like YOLO. This dataset’s challenges, such as varied lighting conditions and differing image resolutions, highlight the limitations of early attempts at grocery item recognition.

George et al.^30^ extended GroZi-120 with the GroZi-3.2k dataset, which comprises 8,350 in-vitro training images and 680 in-situ test images for 80 grocery categories. Notably, the training images have uniform white backgrounds to facilitate multi-label classification. The testing was carried out on a subset of 3,235 images across 27 food classes. Though an improvement over GroZi-120, the dataset still exhibits a significant domain shift and is not well-suited for real-time object detection.

In contrast to web-based datasets, Jund et al.^28^ introduced a real-world dataset containing 4,947 images of 25 grocery products captured in-store using four cameras under diverse conditions. The dataset includes lighting variations, reflections, and shadows, making it more realistic. However, the dataset is limited in scale, both in terms of the number of classes and image types, as it only includes close-shot images with fewer than 10 product instances per image. Figure 2 presents representative samples from the existing datasets.

Goldman et al.^26^ proposed SKU110K, a large-scale dataset focused on densely packed product identification. It contains 11,762 images comprising 110,712 object instances, with all items grouped into a single class labeled “object.” The dataset introduced the concept of long-shot images for grocery product detection and comprises 8,232 training images, 587 validation images, and 2,940 test images. Although not intended for fine-grained classification, SKU110K laid the foundation for understanding object density in detection tasks.

Zhang et al.^27^ developed a retrieval-focused dataset containing 3,153 overlapping shelf images and 200 query images, each with a single target item. Although primarily intended for object retrieval, the dataset can be adapted for detection tasks. However, its narrow focus on a single store and heavy image overlap limits its generalizability.

**Fig. 3:**
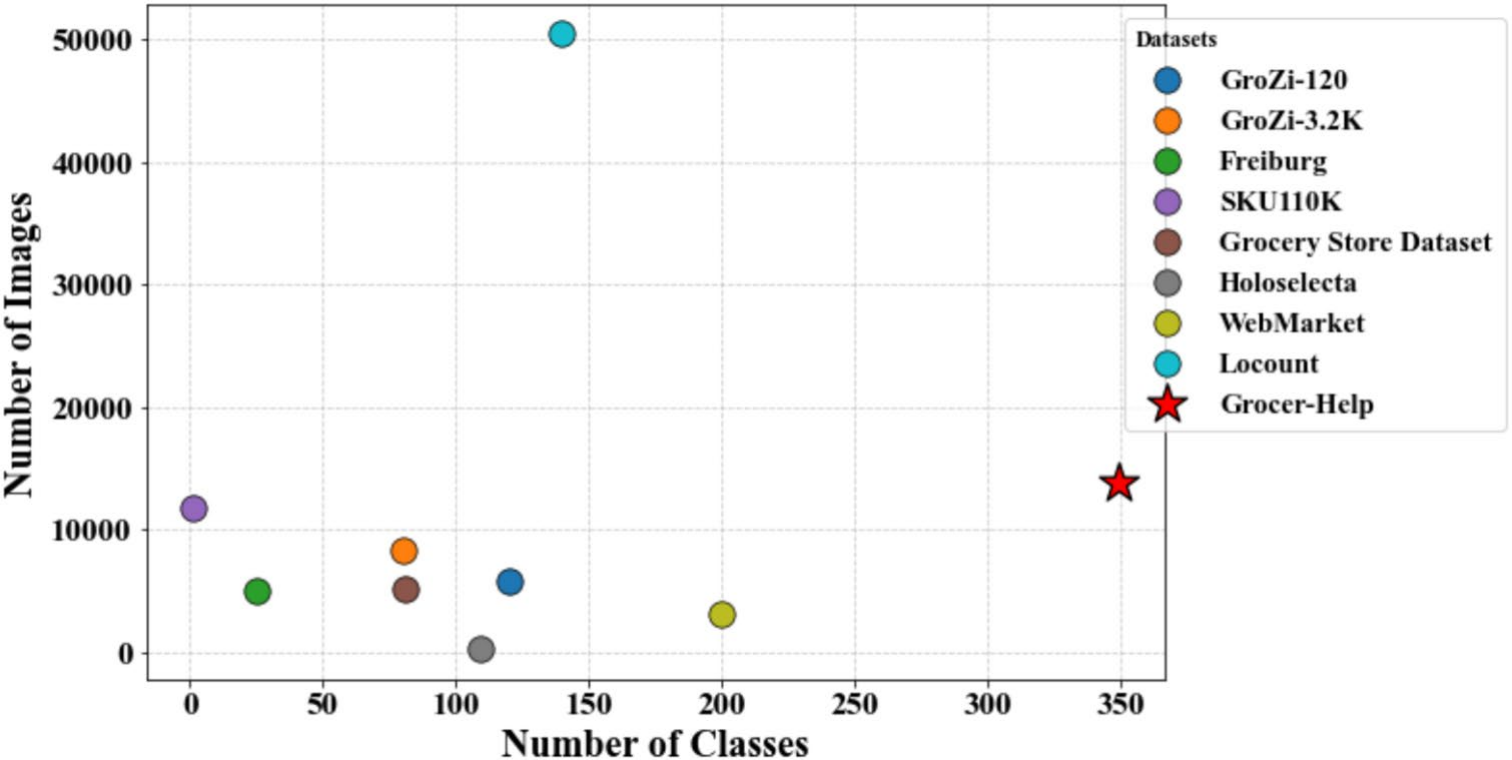
Comparison of the number of images and object classes in the proposed Grocer-Help dataset with existing grocery-related datasets. The figure highlights the scale and diversity of Grocer-Help, demonstrating its comprehensive coverage relative to other publicly available datasets used for grocery product identification.

Klasson et al.^31^ compiled 5,125 close-shot images across 81 product classes collected from 18 different stores. This dataset introduced a hierarchical structure, consolidating the 81 classes into 46 coarse-grained categories. The dataset blends studio-quality and real-world images, capturing products on shelves and in hand-held scenarios. Although effective for identifying individual products, its utility in detecting multiple products in complex scenes is limited.

Fuchs et al.^32^ presented Holoselecta, a dataset designed around vending machine product identification. It includes 295 images featuring 10,035 object instances across 109 classes. The dataset explores structured layouts in vending machines and avoids both close-shot sparsity and dense long-shot complexities. Experimental evaluation was restricted to 39 classes with at least 100 training and 20 test samples each.

Cai et al.^33^ proposed Locount, comprising 50,394 images from 28 different stores. These images span 140 fine-grained classes grouped under nine coarse categories. The dataset includes 34,022 training and 16,372 testing images. While diverse in geographic coverage, all images are captured under similar lighting conditions and are close-shot, limiting their applicability for scenarios involving dense or long-range product arrangements. Figure 3 compares the number of images and classes in the existing datasets with those in our proposed dataset, Grocer-Help.

While existing datasets have made significant strides in grocery product identification, they each come with notable limitations, such as small class counts, homogeneous image types, or constrained domains. Most datasets are either composed of synthetic or close-up images, or suffer from domain mismatches between training and testing. These shortcomings underscore the need for a comprehensive, real-world dataset that captures the diversity of modern retail environments across camera angles, distances, and geographic regions, an objective addressed by our proposed Grocer-Help dataset.

## Proposed Dataset

In this section, we present the proposed grocery identification dataset named Grocer-Help. The dataset is designed to support object detection and classification tasks in real-world retail environments. The images were collected across eight grocery stores, each located in one of five Indian states-Jammu, Uttarakhand, Bihar, New Delhi, Madhya Pradesh, and Karnataka. This geographic diversity contributes to the dataset’s robustness, as grocery stores in different regions often stock a mix of local and international brands, resulting in a wide range of visual appearances and packaging styles. The images were captured under natural conditions from multiple viewpoints, encompassing various lighting settings, occlusions, and shelving layouts. This approach ensures a high degree of variation in spatial arrangement and visual presentation, closely reflecting real-world retail scenarios. Each store’s unique shelving configuration further introduces layout diversity, enhancing the dataset’s generalizability across different environments. The Grocer-Help dataset consists of 13,771 images captured using six different camera devices. Table 2 outlines the technical specifications of these devices.

**Table 2:** Specifications of the camera devices and number of captured images per camera in the Grocer-Help dataset. MP denotes megapixels.

Cam-ID	MP	Aperture	Resolution	Lens Features	# Images
Cam-1	64	f/1.9	4032$\times$2034	Super Wide Angle, Bokeh, Super Macro	1595
Cam-2	64	f/2.4	3456$\times$4608	Ultra Wide Angle, Telephoto, Macro	1792
Cam-3	13	f/2.2	4160$\times$3120	Ultra Wide Angle	2039
Cam-4	48	f/2.0	3000$\times$4000	Ultra Wide Angle, Macro	2748
Cam-5	50	f/2.4	3200$\times$4200	Ultra Wide Angle, Macro	1290
Cam-6	64	f/2.2	4060$\times$3200	Super Wide Angle, Super Macro, Google	2000

**Fig. 4:**
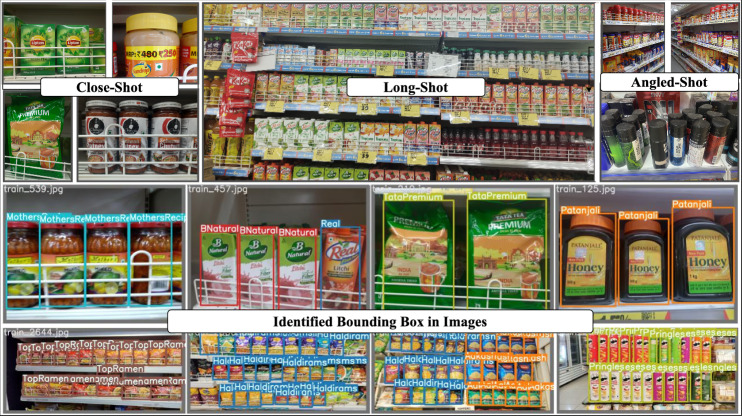
Sample image instances from the proposed Grocer-Help dataset, illustrating the diversity in capture conditions along with corresponding bounding box annotations.

**Fig. 5:**
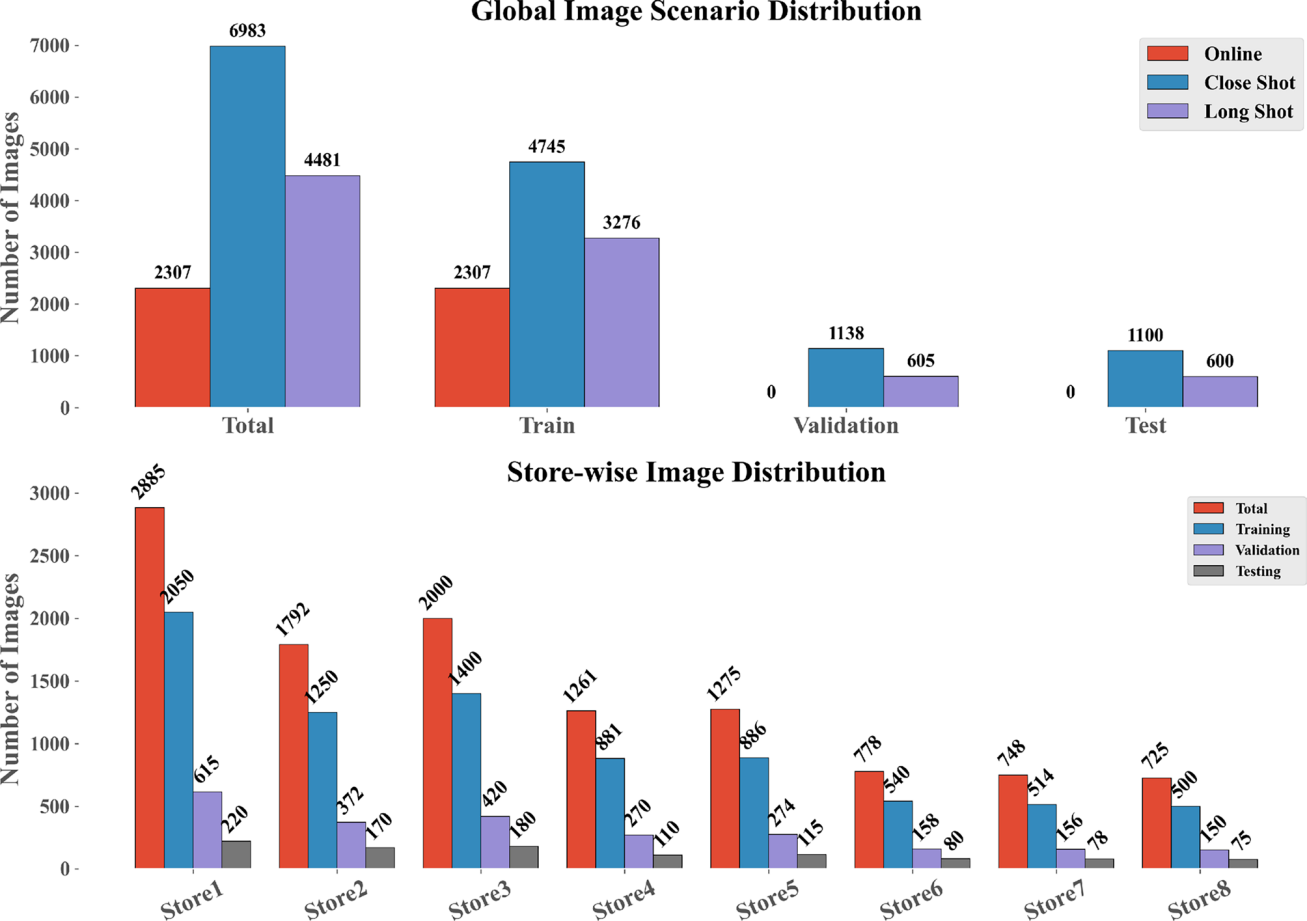
Distribution of image categories within the Grocer-Help dataset, illustrating the proportion of close-shot, long-shot, and online images.

Figure 4 displays representative image samples from the dataset, capturing various visual conditions and product variations. The images contain grocery products from the same class or from multiple different classes. While densely packed scenes are more likely to include a diverse set of categories, a balanced distribution of product variety is maintained across the dataset.

The dataset comprises approximately 4,000 unique grocery products, which have been organized into 349 classes based on brand identity. These classes group together products from the same brand that share consistent visual features such as logo placement, color schemes, and packaging design. This brand-centric classification emphasizes intra-brand visual similarity, allowing machine learning models to leverage brand-level patterns for improved identification performance. Annotations were created using the Roboflow platform, employing bounding boxes to precisely label product instances. Partially visible or blurry products were annotated if key visual characteristics remained identifiable. Figure 5 summarizes the distribution of images across different stores and capture conditions.

The Grocer-Help dataset is structured to capture variation across different image acquisition conditions and is categorized into three groups: Close-Shot (6,983 images), Long-Shot (4,481 images), and Online images (2,307 images collected from e-commerce platforms). In total, the dataset consists of 13,771 images, partitioned into 10,328 for training, 1,722 for validation, and 1,721 for testing. The dataset was collected from eight stores, with each store contributing a varying number of images to reflect realistic retail diversity. For example, Store-1 contributes 2,885 images (2,050 training, 615 validation, and 220 testing), captured using camera Cam-1 and Cam-5. Store-2 provides 1,792 images (1,250 training, 372 validation, and 170 testing) captured using Cam-2, while Store-4 contributes 2,039 images (881 training, 270 validation, and 110 testing) captured using Cam-3. Within the train–validation–test split, the training set contains 4,745 Close-Shot, 3,276 Long-Shot, and all 2,307 Online images. The validation set contains 1,479 Close-Shot and 936 Long-Shot images, while the testing set includes 759 Close-Shot and 269 Long-Shot images. On average, Long-Shot images contain approximately 25 object instances per image, compared to around 9 instances in Close-Shot images, making this dataset suitable for both single-object and dense multi-object detection scenarios. In total, the dataset includes 166,306 annotated object instances, making it one of the largest available benchmarks for grocery product detection in terms of both class diversity and annotation volume. All images were resized to $640 \times 640$ pixels and normalized using standard ImageNet statistics (mean = [0.485, 0.456, 0.406]; std = [0.229, 0.224, 0.225]). To maintain the natural variability and realism of the captured images, no data augmentation was applied during preprocessing.

**Table 3:** Qualitative comparison of the proposed dataset with the existing benchmarks.

Dataset	Visual Diversity	Capture Variability	Illumination Diversity	Scene Complexity	Capture Conditions	Class Granularity
Grozi-120	Low	Low	Low	Medium	Varied	Fine
Grozi-3.2K	Medium	Medium	Low	Low	Varied	Fine
Freiburg	Medium	Low	Low	Low	Homogeneous	Fine
SKU110K	High	Medium	Low	High	Homogeneous	Coarse
Locount	High	High	Medium	High	Varied	Coarse
Holoselecta	Medium	Medium	Low	Medium	Homogeneous	Coarse
**Grocer-Help**	**High**	**High**	**Medium**	**High**	**Varied**	**Fine**

A qualitative evaluation of the proposed Grocer-Help dataset was conducted using six key assessment dimensions: visual diversity, capture variability, illumination diversity, scene complexity, capture conditions, and class granularity. Table 3 highlights the qualitative comparison of the proposed Grocer-Help dataset with the existing benchmark datasets. Visual diversity reflects differences in object positioning, background context, clutter level, and environmental appearance. Capture variability assesses whether images were acquired under multiple capture scenarios, such as close-shot versus long-shot perspectives. Illumination diversity examines variation in lighting conditions, including shadows, natural/artificial illumination, glare, and exposure differences. Scene complexity is measured based on object density, where densely packed scenes indicate higher complexity than sparsely populated ones. Capture conditions account for variations in scale, viewpoint, and angle, reflecting how objects naturally appear in real operational environments. Class granularity evaluates whether categories are fine-grained (e.g., product variants) or coarse-grained (general product type), ensuring the dataset supports real-world recognition demands. Based on these qualitative metrics, the Grocer-Help dataset demonstrates higher environmental realism, variability, and granularity compared to existing benchmark datasets. This enhanced diversity better reflects real-world retail conditions and strengthens its suitability for training and evaluating robust grocery product detection models.

The image collection process was guided by four key considerations to ensure comprehensive coverage of real-world scenarios. First, images were captured from a distance to represent densely populated store shelves, simulating the perspective of surveillance systems such as CCTV cameras. These wide-angle shots help models learn to identify products based on visible patterns from afar. Second, close-up images were captured to highlight detailed and crisp visual features, offering the most distinguishable patterns. These images replicate typical mobile phone captures used for consumer-level identification. Third, catalog-style images were sourced from online platforms. These images present the clearest product visuals, free from background clutter or occlusions, and serve as ideal references for appearance-based learning. Finally, to simulate real-world user behavior, images were taken while in motion, resulting in variations in viewing angles and occasional motion blur. This scenario introduces natural noise into the dataset and trains the model to handle angle distortions and image degradation.

Although the proposed dataset is highly comparable and advances research in this domain, it is not intended to represent all global products. The dataset is inherently biased toward products available in the Indian retail market, and the total number of product categories remains smaller than the vast diversity present in real-world markets. However, its significance extends well beyond being a regional collection. The dataset is carefully designed to capture practical retail complexities, such as densely packed and sparsely arranged shelves, frontal and angled viewpoints, occlusions, and varying real-world conditions. These characteristics make it a valuable resource for evaluating the robustness and generalization ability of deep learning models under realistic deployment scenarios. Rather than serving merely as a regional benchmark, the dataset provides a meaningful testbed for developing and validating real-world object detection algorithms. It lays a foundational framework for autonomous retail intelligence systems and contributes toward the evolution of object detection methods capable of handling complex, real-life environments.

## Methodology

**Fig. 6:**
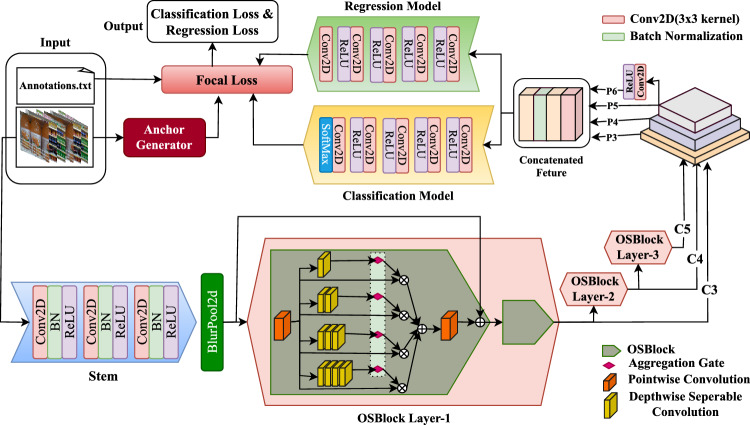
Illustration of the object detection process from input images. The figure demonstrates how an image containing multiple objects is processed by an object detection model to localize, classify, and label each item, producing bounding boxes and class predictions as output.

**Algorithm 1:**
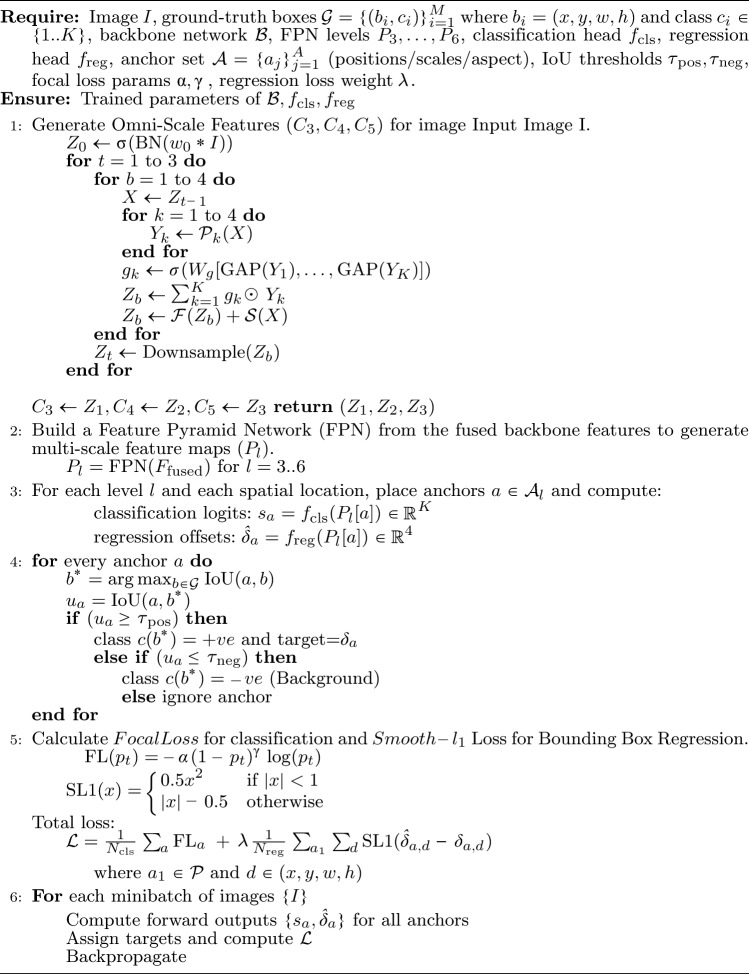
Object detection pipeline.

This section presents the proposed methodology for object detection in visually complex environments, such as grocery store settings. The approach begins with the design of a novel backbone network that serves as a robust feature extractor. These extracted features are subsequently refined using a Feature Pyramid Network (FPN), which captures multi-scale contextual information and enhances feature resolution across different levels. The resulting feature maps are then employed to predict object bounding boxes and their corresponding class labels. To facilitate accurate localization of objects, the model generates nine anchor boxes per spatial location of each feature map level. These anchors are defined using a combination of three aspect ratios [0.5, 1.0, 2.0], which represent tall, square, and wide boxes respectively, and three scale factors [$2^0$, $2^{1/3}$, $2^{2/3}$], corresponding to approximately [1.0, 1.26, 1.59]. This formulation ensures that the anchor set spans a wide range of object shapes and sizes, enabling the detector to effectively match ground-truth objects with varying spatial characteristics across different levels of the feature pyramid. Once predictions are generated, Non-Maximum Suppression (NMS) is applied to eliminate redundant and overlapping bounding boxes. This post-processing step ensures that only the most confident bounding box is retained for each detected object, thereby producing a set of refined and distinct object localizations. The complete model architecture is illustrated in Fig. 6. The OSNet-inspired backbone is designed to generate a more versatile and detection-friendly feature representation. While the overall architecture follows the OSNet paradigm, several key enhancements were incorporated to better suit object detection requirements. First, the original single 7$\times$7 convolutional stem was replaced with a three-stage 3$\times$3 convolution stack, which maintains higher spatial resolution and captures fine-grained edge and texture cues. This modification is particularly beneficial for detecting small objects that may otherwise be lost due to aggressive early downsampling. To further retain structural integrity, we replaced the standard MaxPool operation with BlurPool, which suppresses aliasing while preserving spatial relationships, ultimately improving localization quality. Second, we introduced depthwise seperable deformable convolutions in the deeper stages of the network (OSBlock2 and OSBlock3), corresponding to the C4 and C5 feature levels. Since these stages operate on high-level semantic feature maps with large receptive fields, replacing static convolution with deformable convolution enables the network to dynamically adapt its sampling locations. This improves robustness to scale variations, irregular object shapes, and occlusions, conditions commonly encountered in real-world dense detection environments. Algorithm 1 summarizes the operational flow of the full object detection pipeline.

### Model Backbone

Automatic detection of grocery items presents significant challenges due to the variable density of object arrangements in real-world settings, images may contain densely packed or sparsely distributed items within the same frame. To address this variability effectively, we propose a novel backbone architecture that leverages OSBlock-based channel aggregation^34^ to generate fine-grained feature representations. The backbone processes input image batches of size $640\times 640$ and outputs multi-scale feature maps at three levels: C3, C4, and C5, with corresponding channel dimensions of 256, 384, and 512, respectively. These omni-scale features are subsequently passed through a Feature Pyramid Network (FPN), which integrates features from different semantic depths (C3, C4, and C5) using a combination of upsampling and lateral connections. This fusion results in semantically rich, high-resolution feature maps. The key advantage of integrating FPN into our omni-scale feature extraction pipeline is its ability to capture both intra-scale and inter-scale feature relationships. The fused features are then concatenated and passed through the classification and bounding box regression heads to predict object classes and their corresponding bounding box coordinates. To reduce the number of trainable parameters and enable deployment on edge devices without relying on model quantization, which often leads to a significant drop in accuracy, depthwise separable convolutions are employed. This approach decomposes standard convolutions into a depthwise convolution followed by a pointwise convolution (i.e., a $1\times 1$), thereby significantly reducing computational complexity while preserving model performance.

### OSBlock

The integration of the Omni-Scale Block (OSBlock), originally introduced in OSNet^34^, enables the network to learn rich, omni-scale feature representations, significantly improving object detection performance. The OSBlock is specifically designed to capture features across multiple receptive fields simultaneously, which is crucial in complex visual environments such as grocery stores where object sizes and arrangements vary considerably.

For a given input *x*, the OSBlock computes a residual representation $\overline{x}$ by aggregating outputs from multiple parallel convolutional paths, each operating at a different spatial scale. The final output of the block is defined as:

1 $$\begin{aligned} y=x+\bar{x}, where: \overline{x} = \sum _{t=1}^4 F^t (x), \end{aligned}$$

Here, $F^t(x)$ represents the feature transformation at the t-th scale, realized by stacking a variable number of convolutional layers. This enables the receptive field to expand progressively, from $3\times 3$ up to $9\times 9$, facilitating the capture of both fine-grained and coarse-grained features. The parallel nature of the OSBlock ensures effective multi-scale feature extraction.

To consolidate the outputs from all scale-specific branches, a channel-wise attention mechanism is applied. Each output $x^t$ from the t-th branch is modulated by a learned attention vector $G(x^t)$, yielding the final residual representation:

2 $$\begin{aligned} \overline{x}= \sum _{t=1}^4 G(x^t) \odot x^t \end{aligned}$$

In this expression, $G(x^t)$ is a channel-wise gating function that assigns a unique importance weight to each channel of $x^t$ and $\odot$ denotes the Hadamard (element-wise) product. Unlike scalar attention mechanisms, this channel-level modulation provides finer control over feature selection, enabling the network to emphasize informative features at each scale. The resulting output $\overline{x}$ effectively integrates multi-scale spatial information into a unified, omni-scale representation. For OSBlock2 and OSBlock3 we use depthwise seperable deformable convolutions to learn the spatial adaptability at semantic level. Which means that the backbone becomes sensitive to the different shapes and sizes of objects in the image. A deformable convolution is defined as:

3 $$\begin{aligned} y_c(p_0)=\sum _{p_n\in R} w_c(p_n) . x_c(p_0+p_n+\Delta p_{n,c}) \end{aligned}$$

where, $\Delta p_{n,c}$ is offset predicted by an auxiliary convolution layer, $w_c(p_n)$ are fixed kernel grids, *R* is regular grid $3\times 3$, $p_0$ represents the current spatial location, and $p_n$ is the sampling position relative to $p_0$. The vector generated as a consequence to the lightweight deformable convolutions are $Y_1,Y_2,Y_3, Y_4$, defined as:

4 $$\begin{aligned} Y_k = \mathcal {P}_k (X), \mathcal {P}_k = f^{(k)} (X) \end{aligned}$$

where,

5 $$\begin{aligned} f^{(k)} (X)=f_k(f_{k-1}(...(f_1(X))...)) \end{aligned}$$

## Experimental Details

### Hyperparameter Settings

All experiments were conducted on a system equipped with an NVIDIA GeForce GTX 1080 Ti GPU, using CUDA 10.2 and PyTorch 1.12.1+cu102. The evaluated models were trained and tested on both established benchmark datasets and the newly proposed Grocer-Help dataset to ensure a comprehensive and balanced evaluation. Each model was trained for 150 epochs without employing domain-specific pretrained weights, thereby ensuring a fair comparison across different architectures and datasets. Although the backbone networks utilize standard ImageNet pretraining as part of their generic initialization pipeline, no additional domain-adaptive or task-specific pretraining was incorporated. This design choice was intended to prevent bias toward specific dataset characteristics and to maintain uniform evaluation conditions. Image augmentation was deliberately excluded from the base experimental results. The proposed dataset inherently captures real-world imbalance and practical retail variability. The introduction of synthetic augmentation techniques was observed to introduce artificial pattern noise and alter the natural data distribution. Instead, the dataset preserves authentic variations, including angled viewpoints, skewed captures, occlusions, and densely packed shelf arrangements. This ensures that model performance reflects robustness to genuine environmental complexity rather than artificially generated distortions. By avoiding additional domain adaptation and augmentation in the baseline experiments, the evaluation more accurately measures the inherent generalization capability of each architecture. However, to analyze the impact of augmentation, controlled image augmentation experiments were conducted during the store-wise dataset analysis presented in Section 6. To maintain strict consistency, identical hyperparameters were applied across all models during training and testing. The learning rate was set to 0.001, with a momentum of 0.937 and a weight decay of 0.0005. Input images were resized to $640 \times 640$ pixels. The Intersection over Union (IoU) threshold was fixed at 0.7, the Non-Maximum Suppression (NMS) threshold at 0.4, and a maximum of 300 detections per image was permitted. This standardized configuration ensures reliable cross-model comparisons and guarantees that observed performance differences arise from architectural characteristics rather than hyperparameter tuning or dataset-specific optimization.

### Loss Function

In this work, Focal Loss is employed as the classification loss function to address the inherent class imbalance. Proposed by Lin et al.^12^ focal loss modifies the standard cross-entropy loss by introducing a modulating factor that down-weights the loss assigned to well-classified examples, thereby focusing training on hard, misclassified instances. The focal loss is formulated as:

6 $$\begin{aligned} FL(p_t)= -\alpha _t(1-p_t)^\gamma log(p_t). \end{aligned}$$

denotes the model’s estimated probability for the ground-truth class, $\alpha _t$ is a weighting factor used to balance the importance of positive/negative samples, and $\gamma$ is a focusing parameter that reduces the contribution of easy examples. In our implementation, we adopt the default settings used in the original RetinaNet, with $\alpha =0.25$ and $\gamma =2.0$, which have been shown to be effective in improving detection performance, particularly in the presence of dense background clutter or class imbalance.

### Evaluation Metric

To evaluate the performance of the proposed object detection model, we employ Precision, Recall, and Mean Average Precision (mAP) at two different Intersection over Union (IoU) thresholds, mAP@0.50 and mAP@0.50–0.95, as our primary evaluation metrics. These metrics are widely regarded as the most informative and reliable for object detection tasks because they provide a comprehensive assessment of both classification correctness and localization accuracy.

Precision measures the proportion of correct positive detections (true positives) among all predicted positives, indicating how well the model avoids false positives. It is computed as:

7 $$\begin{aligned} Precision = \frac{TP}{TP + FP} \end{aligned}$$

where, TP and FP denote the number of true and false positives, respectively.

Recall, on the other hand, quantifies the model’s ability to detect all relevant objects by computing the proportion of ground-truth instances that are correctly detected:

8 $$\begin{aligned} Recall = \frac{TP}{TP + FN} \end{aligned}$$

where, FN is the number of false negatives.

Mean Average Precision (mAP) is the standard metric for object detection. For a given class, the Average Precision (AP) is calculated as the area under the precision–recall curve. The mean of APs across all classes gives the final mAP score. The use of IoU thresholds (e.g., 0.5 and 0.9) allows for evaluation under both lenient and strict matching criteria, where mAP@0.50 considers a detection correct if the IoU between the predicted and ground-truth box is at least 0.5, providing a measure of coarse localization performance and mAP@0.50–0.95 applies a much stricter criterion, requiring almost perfect overlap, and thus reflects the model’s fine-grained localization ability. By evaluating performance using both precision–recall metrics and mAP at multiple IoU thresholds, we ensure a balanced and rigorous assessment of the detector’s capability to identify, classify, and localize objects accurately under real-world conditions.

## Results & Analysis

###  Baseline Analysis

The experimental results presented in Table 4 report the performance of state-of-the-art object detection models in terms of mAP@0.50 and mAP@0.50–0.95 across various benchmark datasets.

**Table 4:** Performance comparison of existing object detection models and the proposed Omni-Scale Feature Model on benchmark grocery datasets. The table reports mAP@0.50 and mAP@0.50–0.95 scores, along with the number of model parameters (Params) and computational complexity in GFLOPs.

Model	Params	FLOPs	GroZi-120		**G_Store**		**GroZi-3.2K**		**Freiburg**		**SKU110K**	
	(M)	(G)	0.50	0.95	0.50	0.95	0.50	0.95	0.50	0.95	0.50	0.95
YOLOv3^5^	103.82	283.7	34.1	22.9	39.4	29.2	37.1	27.1	88.1	45.2	91.4	59.8
YOLOv5m^6^	25.17	64.9	23.9	12.1	29.2	18.8	26.1	16.8	83.0	39.9	90.5	58.4
YOLOv7^4^	38.14	108.1	25.6	13.2	31.2	20.1	27.5	16.9	79.4	39.7	88.5	51.1
YOLOv8m^7^	25.96	79.6	25.2	12.9	31.8	19.9	26.7	16.6	82.4	43.5	90.9	59.2
YOLOv9m^8^	20.28	78.3	26.3	13.6	32.1	20.6	29.1	17.2	83.5	45.1	92.2	58.9
YOLOv10m^9^	16.68	65.1	21.7	9.5	22.8	10.5	20.2	10.3	73.7	46.0	82.1	60.0
YOLOv11m^10^	20.18	68.9	27.3	13.3	32.9	21.0	29.7	17.1	84.0	46.1	92.4	60.1
YOLOv26m^11^	22.04	76.2	28.1	17.4	34.3	23.7	33.7	22.3.1	88.2	60.1	92.7	62.3
RetinaNet^12^	39.96	72.1	24.1	–	31.3	–	26.6	–	78.9	–	90.0	–
RtDETR-x^14^	67.66	233.0	15.6	7.2	18.8	7.8	15.7	6.1	52.9	21.8	67.9	38.1
**Proposed Model**	**14.97**	**116.0**	**25.8**	**17.4**	**28.3**	**17.2**	**25.9**	**16.6**	**82.7**	**41.5**	**91.2**	**55.6**

G_Store: Grocery Store Dataset.

Among these, SKU110K consistently achieves the highest mAP scores across all models, while Grozi-120 yields the lowest performance. YOLOv3 emerges as the best-performing model across datasets, demonstrating strong detection capability for grocery items, whereas RT-DETR-x records the lowest mAP scores, indicating its relative limitations in handling the complexity of grocery identification tasks. A closer examination of the results reveals plausible explanations for these trends. The superior performance on SKU110K can be attributed to its single-class structure, which simplifies the classification task and contributes to higher mAP values. In contrast, Grozi-120 demonstrates lower performance due to a significant domain shift between the training and testing sets, which negatively impacts model generalization.

Table 5 presents the results of our proposed Grocer-Help dataset across the evaluated models. Despite containing a significantly larger number of classes than SKU110K, the Grocer-Help dataset achieves competitive mAP values, indicating a well-balanced and representative design. Compared to Grozi-120, Grocer-Help offers better generalizability and consistency, supporting its effectiveness for real-world grocery identification scenarios. Furthermore, our proposed Omni-Scale Feature Model demonstrates competitive performance with considerably fewer parameters. While YOLOv3 remains the top-performing baseline model, our model achieves nearly equivalent mAP scores across datasets with a reduced parameter count. Specifically, when compared to YOLOv10-m, which has 16.68 million parameters, our model achieves superior performance on most benchmark datasets using only 14.97 million parameters, underscoring its computational efficiency and practical utility.

### Dataset Generalizability

The results in Table 5 present the performance of various state-of-the-art object detection models, along with the proposed Omni-Scale Feature Model, evaluated on the newly introduced Grocer-Help dataset. Despite the inherent challenges posed by the dataset, such as a large number of object classes and significant domain variation, existing models demonstrate stable performance, confirming the dataset’s robustness and practical applicability.

**Table 5:** Performance of existing object detection models and the proposed Omni-Scale Feature Model on the newly introduced Grocer-Help dataset. The table reports key evaluation metrics including mAP@0.50, mAP@0.50–0.95, precision, and recall, alongside the number of parameters (Params) and computational complexity (GFLOPs).

Model	Params (M)	FLOPs (G)	mAP@0.50 (%)	mAP@0.50–0.95 (%)	Precision (%)	Recall (%)
YOLOv3^5^	103.82	283.7	64.8	50.3	63.0	59.1
YOLOv5m^6^	25.17	64.9	61.1	46.5	65.3	55.1
YOLOv7^4^	38.14	108.1	52.8	13.0	62.8	67.9
YOLOv8m^7^	25.96	79.6	61.1	40.0	68.3	46.0
YOLOv9m^8^	20.28	78.3	56.2	43.7	73.4	48.6
YOLOv10m^9^	16.68	65.1	39.8	23.6	64.8	25.9
YOLOv11m^10^	20.18	68.9	58.2	42.0	71.1	46.3
YOLOv26m^11^	22.04	76.2	59.1	46.3	62.9	52.4
RetinaNet^12^	39.96	72.1	56.2	–	63.6	55.1
RtDETR-x^14^	67.66	233.0	19.1	15.3	31.7	15.8
**Proposed Model**	**14.97**	**116.0**	**58.3**	**21.3**	**72.9**	**77.9**

Among the evaluated models, YOLOv3 achieves the highest mAP@0.50 score of 64.8, indicating that, on average, the model correctly identifies objects with a good balance of precision and recall at an Intersection over Union (IoU) threshold of 0.5. In this setting, YOLOv3 records a precision of 63.0 and a recall of 59.1, suggesting that 63% of its predictions are correct and it successfully detects approximately 59% of the actual objects. In contrast, the proposed Omni-Scale Feature Model attains a slightly lower mAP@0.50 compared to YOLOv3; however, it achieves significantly higher precision and recall, with 73% of predictions being correct and a greater proportion of true objects being identified. This indicates that the proposed model is capable of generating a broader range of valid detections, with higher accuracy in identifying true positives. The relatively lower mAP@0.50, despite improved precision and recall, suggests that the proposed model is more inclusive in generating potential object detections, which may lead to slightly reduced localization accuracy at the 0.5 IoU threshold. Nevertheless, the overall detection quality remains strong, underscoring the model’s effectiveness in identifying a diverse set of products in complex retail environments

**Fig. 7:**
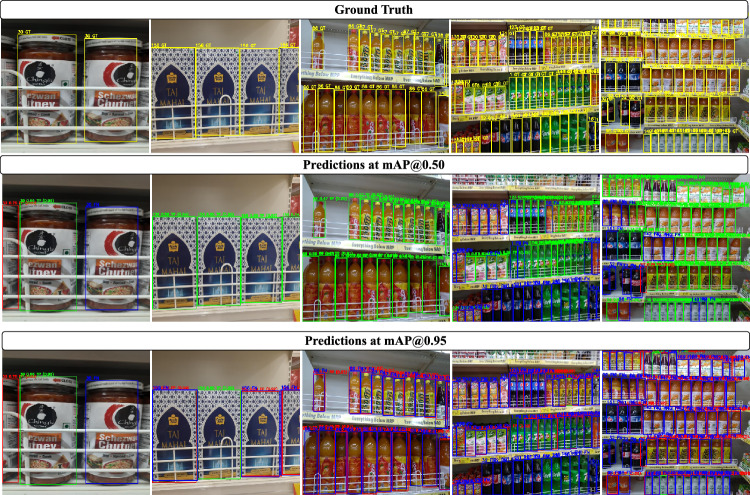
Images showing objects annotated with ground truth (GT) bounding boxes and predicted detections at IoU thresholds mAP@0.5 and mAP@0.95. This illustrates the model’s detection performance across varying scenarios.

The results presented in Table 5 indicate that while the proposed model outperforms existing approaches in terms of mAP@0.5, its performance at mAP@0.95 is significantly lower. This drop can be attributed to several factors. Primarily, the proposed backbone emphasizes recall and semantic richness, which improves object detection coverage but can compromise localization precision. As a result, the model is less accurate in tightly bounding objects, especially in densely populated scenes. Visual examples of these prediction cases are illustrated in Fig. 7. Another contributing factor is the aggregation gate in the OSBlock, which fuses features across multiple scales to generate richer embeddings but can reduce spatial accuracy at stricter IoU thresholds. Additionally, the inherent heterogeneity of the Grocer-Help dataset, including the presence of very small objects, further challenges precise localization, resulting in lower mAP scores at higher IoU thresholds (0.50–0.95). We intend to address this dip in mAP accuracy in future work.

**Fig. 8:**
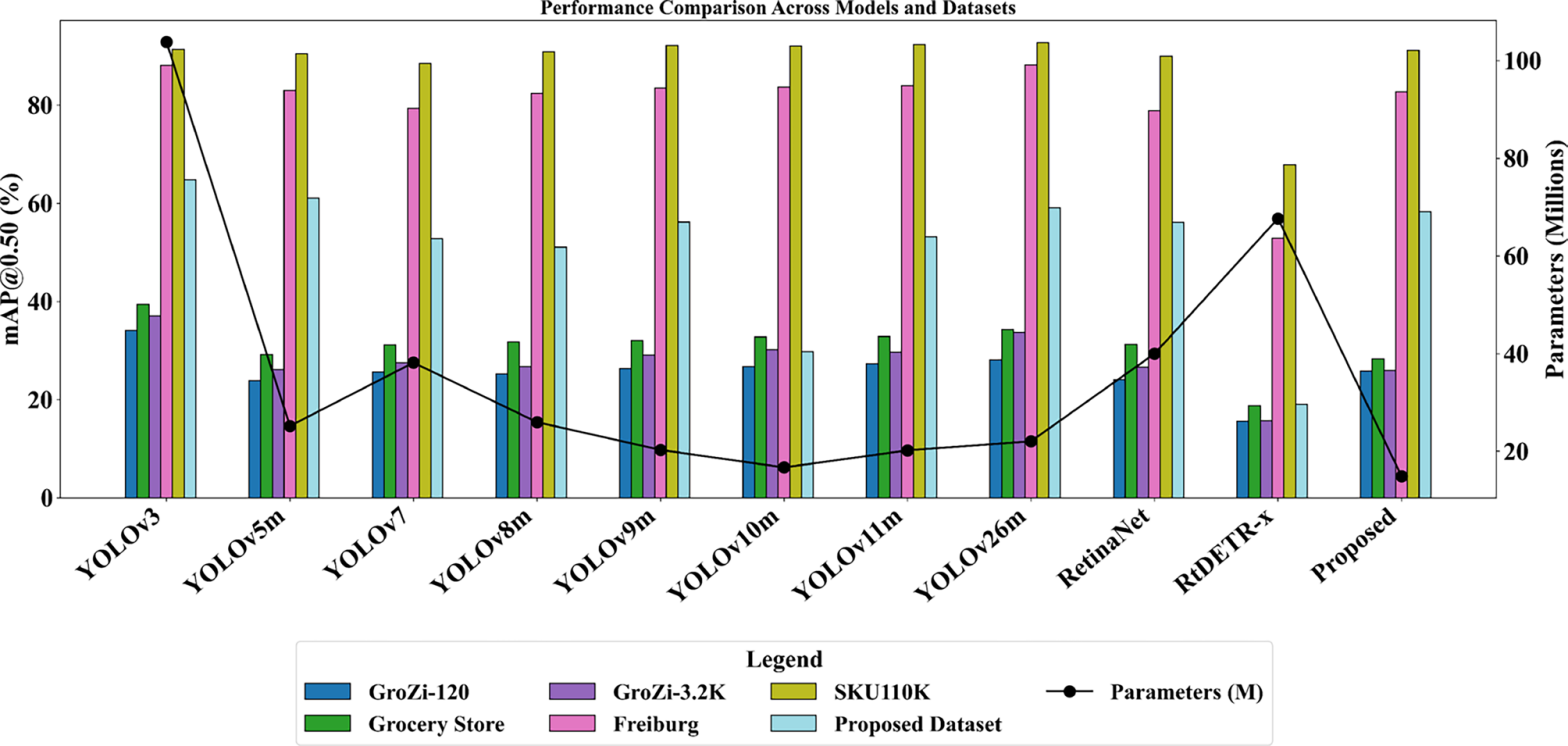
Comparative analysis of mAP@0.50 and mAP@0.50–0.95.50.95 achieved by various object detection models across multiple benchmark datasets, including the proposed Grocer-Help dataset.

**Fig. 9:**
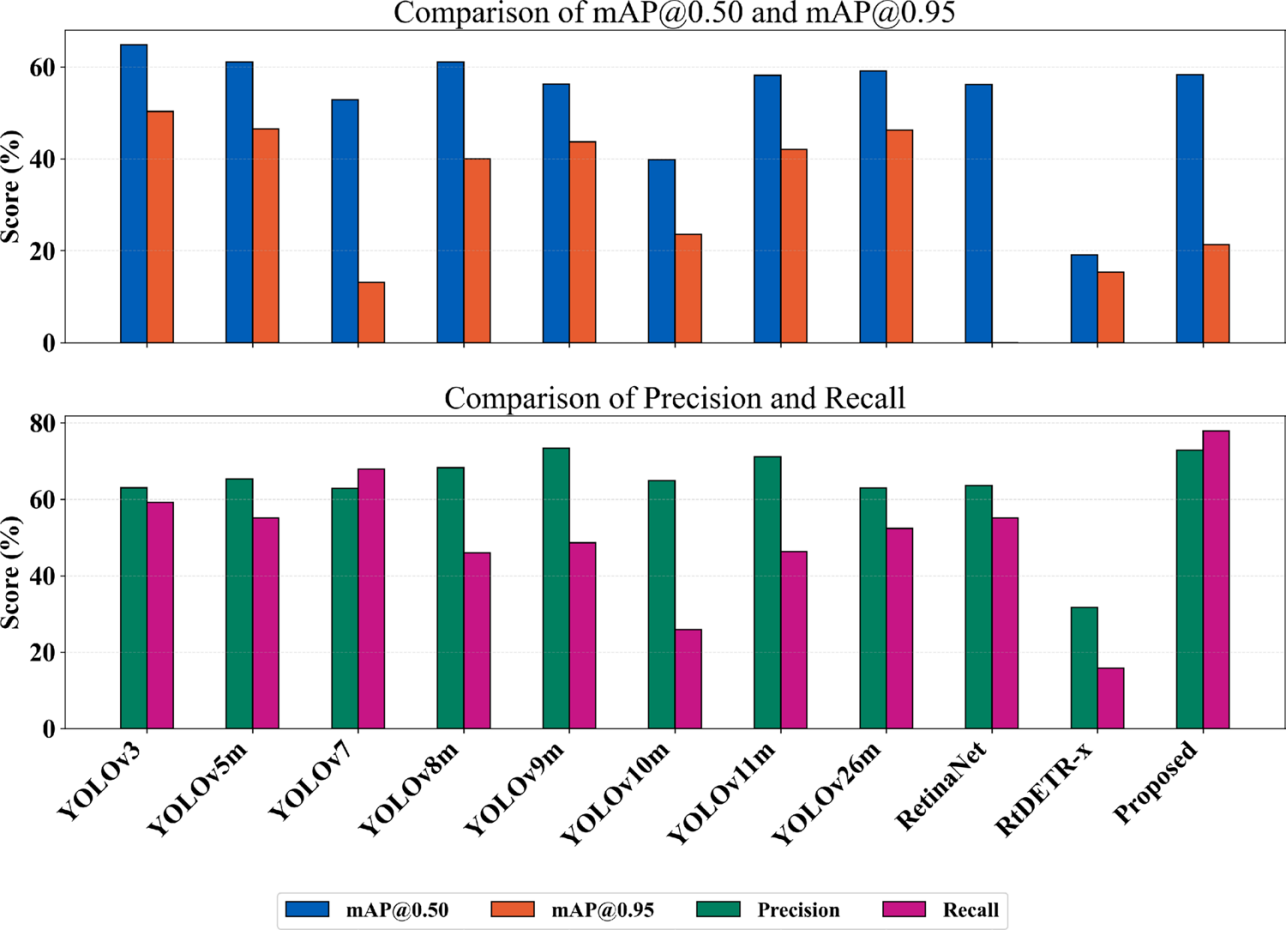
Performance comparison of various object detection models on the proposed Grocer-Help dataset.

Figure 8 provides a comprehensive visual representation of the results summarized in Table 4 and Table 5. The figure demonstrates that the proposed Omni-Scale Feature Model performs competitively with state-of-the-art models and, in several cases, surpasses them while utilizing fewer parameters. Additionally, the figure highlights the strong performance of the proposed Grocer-Help dataset, which compares favorably with existing benchmark datasets across multiple models.

Figure 9 summarizes the results presented in Table 5, visually demonstrating that the proposed model performs competitively in terms of mAP@0.50 compared to existing models, while significantly outperforming them in terms of precision and recall. To statistically evaluate the proposed model, we randomly sampled 800 images from the full test set of 1,700 images. Five independent random subsets were generated in this manner, resulting in five distinct test folds. Each fold therefore represents a different subset of the test data and may contain a varying number of object classes depending on the sampled images. For statistical comparison, we selected YOLOv11, the next lightest model, as the baseline. Both models were evaluated on each of the five test folds, and their performances were compared using the Wilcoxon signed-rank test. This paired non-parametric test was conducted to assess whether the observed performance differences across the folds were statistically significant. The results, summarized in Table 6, demonstrate consistent performance gains of the proposed model over YOLOv11 across the sampled test folds.

**Table 6:** Statistical comparison between the proposed model and YOLO11m across five independent test iterations.

Test Fold	Model	mAP@0.50	Std	Median AP	p-value
Fold-1	YOLOv11m	0.575	0.25	0.51	-
	**Proposed**	0.598	0.27	0.51	0.036
Fold-2	YOLOv11m	0.552	0.26	0.52	-
	**Proposed**	0.581	0.25	0.51	0.035
Fold-3	YOLOv11m	0.554	0.23	0.51	-
	**Proposed**	0.579	0.24	0.52	0.036
Fold-4	YOLOv11m	0.551	0.21	0.52	-
	**Proposed**	0.572	0.22	0.51	0.036
Fold-5	YOLOv11m	0.58	0.22	0.53	-
	**Proposed**	0.60	0.23	0.54	0.037

To the best of our knowledge, the proposed dataset offers a unique experimental scenario not previously explored in existing research. By incorporating three distinct domains of images within a single dataset–close-shot, long-shot, and online catalog images, it enables a detailed analysis of the effects of product density and capturing angles on model performance. Table 7 illustrates the impact of varying image capture distances on object detection and classification accuracy.

**Table 7:** mAP@0.50 and mAP@0.50–0.95 results across different training and testing combinations using the Grocer-Help dataset. The scenarios include various combinations of close-shot, long-shot, and online images to evaluate the impact of image type variability on model performance in real-world settings.

# Classes	Train	Test	Training Scenario	Train Instance	Testing Scenario	Test Instance	mAP@ 0.5	mAP@ 0.9
251	3276	269	LongShot	77039	LongShot	6836	53.30	24.79
240	4745	759	CloseShot	36445	CloseShot	6747	61.65	27.30
240	3276	759	LongShot	77039	CloseShot	6747	52.00	17.10
251	4745	269	CloseShot	36445	LongShot	6836	22.03	10.47
251	8021	269	CloseShot, LongShot	113484	LongShot	6836	63.03	25.47
240	8021	759	CloseShot, LongShot	113484	CloseShot	6747	64.70	21.60
349	8021	1028	CloseShot, LongShot	113484	CloseShot, LongShot	13583	64.90	29.10
251	10328	269	LongShot, CloseShot, Online	115791	LongShot	6836	57.32	15.92
240	10328	759	LongShot, CloseShot, Online	115791	CloseShot	6747	63.01	24.61
349	10328	1028	LongShot, CloseShot, Online	115791	LongShot, CloseShot	13583	58.32	21.31

### Diversity performance analisys

The results presented in Table 7, obtained using the proposed model on the Grocer-Help dataset, indicate that the best performance is achieved when both training and testing are conducted on close-shot images. This is expected, as close-shot images provide clearer and more prominent visual features, making object recognition more reliable. In contrast, the model performs worst when trained on close-shot images and tested on long-shot images. In this scenario, the reduced resolution and increased object density in long-shot images lead to misclassifications, where multiple objects may be incorrectly grouped and identified as a single class. Overall, the analysis shows that testing on close-shot images–regardless of the training domain–consistently results in higher mAP@0.50 values compared to test scenarios involving long-shot images.

### Store-wise performance analysis

To further evaluate the robustness of the proposed dataset, we conducted a store-wise analysis of the eight participating stores. The objective was to investigate whether the model’s detection accuracy varies with data captured from different store environments.

**Table 8:** Store-wise performance analysis on the proposed Grocer-Help dataset highlighting the impact of store-specific characteristics on detection accuracy.

# Classes	Store	Train Images	Test Images	Augmentation	mAP @0.5	mAP @0.9
176	*I*	2050	220	None	74.34	60.46
		8200	220	C.S, Flip, Noise	74.20	58.01
231	*II*	1250	170	None	70.77	62.62
		5000	170	C.S, Flip, Noise	72.61	62.49
207	*III*	1400	180	None	62.87	41.21
		5600	180	C.S, Flip, Noise	64.18	41.34
181	*IV*	881	110	None	64.24	40.21
		3524	110	C.S, Flip, Noise	66.20	40.34
178	*V*	886	115	None	65.18	45.54
		3544	115	C.S, Flip, Noise	68.02	45.55
157	*VI*	540	80	None	62.44	41.12
		2160	80	C.S, Flip, Noise	63.20	45.50
98	*VII*	514	78	None	71.31	59.17
		2056	78	C.S, Flip, Noise	74.14	61.73
106	*VIII*	500	75	None	63.45	40.83
		2000	75	C.S, Flip, Noise	64.20	42.64

**C.S:** Contrast-Saturation Augmentation, **Flip:** Horizontal & Vertical flip, **Noise:** Salt and Pepper noise.

**Fig. 10:**
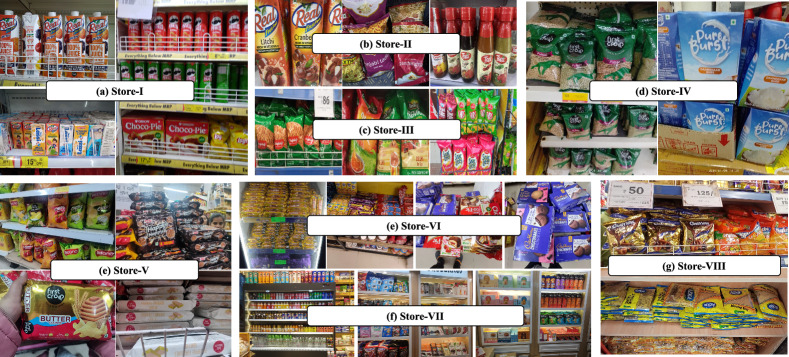
Sample image instances from three selected stores in the Grocer-Help dataset, organized store-wise. The images reflect variations in product arrangement, lighting conditions, and packaging styles across different retail environments, providing insights into store-specific visual diversity.

Since the number of images per store was insufficient for effective training with deep neural networks, we employed contrast-saturation, horizontal and vertical flip, and salt and pepper noise augmentation was to increase the sample size. This also allowed us to examine the impact of augmentation in a complex, real-world setting. The results, presented in Table 8, indicate that the model exhibits varying mAP scores across different stores, contrary to our initial assumption that a single model would generalize equally well across all store environments. However, the variation in performance is relatively modest. Interestingly, the augmentation had minimal impact on mAP in most cases, with the exception of Store-1, where it led to a minute decrement.

This finding prompted a qualitative review of the dataset. Upon analysis, we observed that Store-1 maintained a more organized product arrangement and featured a higher proportion of items with box packaging, which likely contributed to the enhanced model performance. These qualitative differences are visually illustrated in the sample images shown in Fig. 10.

**Table 9:** Impact of training instance frequency on model prediction accuracy for the Grocer-Help dataset. The table presents mAP@0.50 and mAP@0.50–0.95 scores across different combinations of training and testing scenarios involving close-shot, long-shot, and online images. Here, Training Instances (T.I) refer to the number of times a grocery product appears in the training set, influencing the model’s ability to accurately detect and classify that product.

Training Scenario	Train Instance	Testing Scenario	Test Instance	Metric	$T.I>1K$	$1K>T.I>500$	$T.I<500$
LongShot	77039	LongShot	6836	mAP@0.50	71.62	66.50	30.10
				mAP@0.50–0.95.50.95	30.20	25.50	7.80
LongShot	77039	CloseShot	6747	mAP@0.50	48.20	30.65	12.23
				mAP@0.50–0.95.50.95	12.00	9.14	5.78
CloseShot	36445	LongShot	6836	mAP@0.50	54.32	41.23	18.34
				mAP@0.50–0.95.50.95	24.32	20.56	10.32
CloseShot	36445	CloseShot	6747	mAP@0.50	83.20	71.20	41.00
				mAP@0.50–0.95.50.95	40.40	32.20	15.90
CloseShot	113484	CloseShot	13583	mAP@0.50	76.90	59.23	24.32
+ LongShot		+ LongShot		mAP@0.50–0.95.50.95	38.30	27.23	9.30

### Instance based analysis

The results presented in Table 9 clearly demonstrate that the number of training instances per product is a critical factor in product identification accuracy. When the number of instances exceeds 1,000, the model achieves significantly higher accuracy. However, a sharp decline in performance is observed when the instance count falls below 500, indicating a threshold effect. This pattern remains consistent across all training and testing scenarios. While the quantity of training data positively impacts prediction accuracy, it is not the only influencing factor. The type of image–such as close-shot versus long-shot–also plays a substantial role. For instance, long-shot images typically include more object instances per frame, yet their prediction performance is often comparable to close-shot images with fewer instances. Notably, when training instances are very limited, the model frequently misclassifies or fails to detect the product, regardless of image type.

## Limitations and future work

While the proposed dataset and model provide several notable advantages, there are also certain limitations that warrant attention. This section outlines key limitations and presents possible directions for future work to address them:

1. **Imbalance in Packaging Types:** The dataset is inherently heterogeneous, leading to an unequal distribution between box-packaged and packet-packaged products. This imbalance can cause skewed detection accuracy, particularly in cases where multiple products exhibit similar visual patterns and low inter-class variation. In real-world scenarios, such visual overlaps may result in frequent misclassifications. *Future Work:* To mitigate this, we propose incorporating an ensemble strategy that combines object detection with text recognition models. While the detection model localizes product regions, a text recognition module can be used to classify products based on label information. This multimodal approach is a promising avenue we intend to explore in future research.
2. **Stacked Product Visibility:** When grocery items are stacked horizontally–such as one on top of the other–visibility of product features is significantly reduced, making identification challenging. Vertical placement of products, where the front face is more prominently visible, tends to yield better detection results. *Future Work:* To address this limitation, we suggest integrating a QR code-based mechanism, where horizontal rows of products are associated with distinct QR tags. This would support improved detection in cases where visual cues are occluded due to horizontal stacking.
3. **Lack of Quantity-Based Annotations:** The current dataset does not differentiate between size or quantity variants of the same product. This limitation arises primarily from the dense nature of the shelf images, where quantity indicators (e.g., weight or volume) are often not visible. *Future Work:* In subsequent work, we aim to incorporate quantity-based categorization by leveraging contextual cues such as relative positioning on rack shelves or using optical character recognition (OCR) to extract size-related text, where visible.
4. **Reduced Accuracy at High IoU Thresholds:** Although the proposed model achieves high detection accuracy at a 0.50 Intersection-over-Union (IoU) threshold, its performance diminishes at higher thresholds (e.g., 0.95). This suggests that while the model is effective for coarse localization, it struggles with tighter bounding box predictions. *Future Work:* We plan to investigate a dynamic anchor-based approach to improve localization precision and boost mAP@0.50–0.95. Adaptive anchor strategies can better handle object scale variations and improve detection alignment for tightly packed scenes.
5. **Reduced Accuracy with inclusion of Online images in training:** Although the proposed model demonstrates competitive accuracy across various training and testing configurations, as shown in Table 7, we observed a noticeable decline in performance when online images were included in the training set. This reduction can be attributed to the visual domain gap between online and in-store images. Online images typically exhibit cleaner backgrounds, controlled lighting, and curated viewpoints, whereas in-store images contain clutter, variable illumination, occlusions, and diverse capture angles. This domain shift reduces the model’s generalization capability and leads to a drop in detection accuracy. *Future Work:* To address this limitation, we plan to explore domain adaptation techniques that can better bridge the gap between these visual domains and improve performance under mixed-domain training conditions.

## Conclusion

This paper introduces Grocer-Help, a comprehensive grocery identification dataset tailored to real-world retail scenarios in supermarkets. The dataset comprises diverse images of daily grocery products captured under varying conditions, including different camera distances, angles, lighting, and motion blur, to systematically address these practical challenges. The images are collected using mobile cameras without requiring explicit camera calibration, as the system is designed to operate on ready-to-capture images from varying focal points and viewpoints. Since the training data inherently includes such variations, the model learns to generalize across different capture conditions without relying on fixed camera parameters. By including data from eight geographically and visually distinct stores, the dataset enables a deeper understanding of store-wise domain variation, which has a measurable impact on detection performance. Although the stores are assumed to be reasonably well-lit, as is typical in supermarket environments, extreme low-light or heavy shadow conditions may still affect model performance during deployment. The dataset permits partial occlusion to reflect practical retail scenarios; however, severe occlusion exceeding approximately 60% of the object area may reduce detection reliability. Furthermore, because supermarket shelves are periodically rearranged, images from multiple stores with varying shelf layouts were intentionally incorporated to simulate realistic shelf re-stacking and product repositioning patterns. Additionally, it incorporates online product images, offering insights into how digital catalog visuals influence object detection accuracy. A major contribution of Grocer-Help lies in its ability to facilitate novel experimental analyses, such as the effect of training instance frequency (i.e., how often a product appears during training) on model accuracy, an aspect previously unexplored due to the limitations of existing datasets. It also highlights how packaging type, particularly plastic sachets, affects detection precision, revealing a new dimension in grocery identification research. Alongside the dataset, we propose a lightweight Omni-Scale Feature Model, which achieves competitive detection accuracy across all datasets, especially on Grocer-Help, while maintaining a significantly lower parameter count compared to state-of-the-art models. This balance of accuracy and computational efficiency makes the model well-suited for real-time deployment in resource-constrained environments.

In summary, Grocer-Help not only sets a new benchmark for grocery product identification but also serves as a valuable resource for studying domain generalization, data imbalance, and visual variation in retail environments. In future work, we plan to expand the dataset with more classes and instances and explore advanced techniques such as object-wise regional attention for dynamic product recognition. We also envision developing a system for automated catalog generation directly from shelf images, streamlining the inventory management and product listing process for retailers.

## Data Availability

The Grocer-Help dataset used in this study is currently part of ongoing research and is not yet fully public. However, to support reproducibility and further research, a portion of the dataset has been made available. The following two links provide access to a representative subset of the images used in our experiments. The complete dataset may be shared upon reasonable request, subject to institutional approval and research progress. https://doi.org/10.5281/zenodo.10464054; https://dx.doi.org/10.21227/d4na-7e68.
